# A Comparison of Cell-Cell Interaction Prediction Tools Based on scRNA-seq Data

**DOI:** 10.3390/biom13081211

**Published:** 2023-08-02

**Authors:** Zihong Xie, Xuri Li, Antonio Mora

**Affiliations:** 1 Joint School of Life Sciences, Guangzhou Medical University and Guangzhou Institutes of Biomedicine and Health (Chinese Academy of Sciences), Guangzhou 511436, China; 2020219480@stu.gzhmu.edu.cn; 2 State Key Laboratory of Ophthalmology, Zhongshan Ophthalmic Center, Sun Yat-Sen University, Guangdong Provincial Key Laboratory of Ophthalmology and Visual Science, Guangzhou 510060, China

**Keywords:** cell-cell interactions (CCIs), single-cell RNA-seq (scRNA-seq), idiopathic pulmonary fibrosis (IPF), CellPhoneDB, NATMI, scMLnet, CellChat, iTALK, SingleCellSignalR, LIANA

## Abstract

Computational prediction of cell-cell interactions (CCIs) is becoming increasingly important for understanding disease development and progression. We present a benchmark study of available CCI prediction tools based on single-cell RNA sequencing (scRNA-seq) data. By comparing prediction outputs with a manually curated gold standard for idiopathic pulmonary fibrosis (IPF), we evaluated prediction performance and processing time of several CCI prediction tools, including CCInx, CellChat, CellPhoneDB, iTALK, NATMI, scMLnet, SingleCellSignalR, and an ensemble of tools. According to our results, CellPhoneDB and NATMI are the best performer CCI prediction tools, among the ones analyzed, when we define a CCI as a source-target-ligand-receptor tetrad. In addition, we recommend specific tools according to different types of research projects and discuss the possible future paths in the field.

## 1. Introduction

Cell-cell interactions (CCIs) refer to biochemical or physical interactions between cells, which play a significant role in the function, development, and homeostasis of multicellular organisms. They include both structural interactions (such as cell adhesion processes and cell-extracellular matrix interactions) and cell-cell communication processes. In cell-cell communication, a ligand secreted by one cell (for example, growth factors, chemokines and cytokines) binds a receptor molecule of another cell and triggers signaling cascades able to alter gene expression [1]. Cell-cell communication can occur through different mechanisms, including autocrine, paracrine, juxtacrine, and endocrine signaling. Autocrine signaling refers to the case where a cell secretes a ligand that binds to a receptor on its own surface. In paracrine signaling, a “source cell” secretes a ligand that reaches a nearby “target cell” and binds to one of its receptors. Juxtacrine signaling refers to cell interactions where cells physically interact through the formation of cell junctions that allow them to share molecules, ions, and electrical signals. Endocrine signaling involves molecules such as hormones that are shared between different organs through the bloodstream. The study of CCIs has proven important in understanding biological mechanisms such as cell differentiation and development [2], tissue and organ homeostasis [3], and multiple areas of disease research, such as immune interactions in the response to disease [4] or the effects of aging, infection, and injuries on multicellular organization [1]. Our own studies have found that neovascularization induced by PDGF-D overexpression in mouse retinal pigment epithelial cells is linked to an increase in CCIs, which may have implications for the treatment of neovascular disease [5].

**Figure 1:**
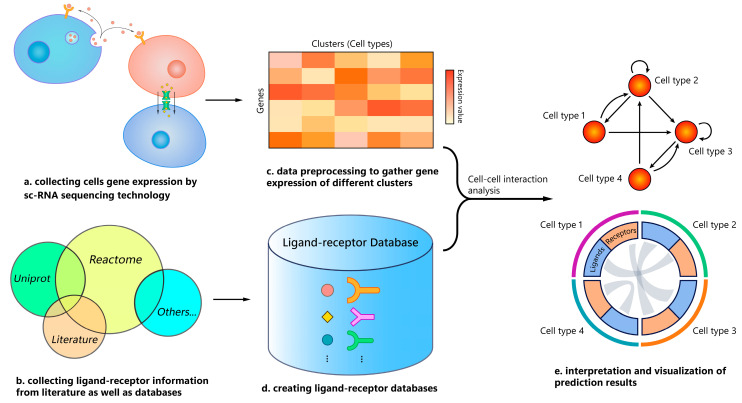
General workflow of cell-cell interaction prediction using single-cell RNA-seq data. (**a**,**c**) Samples are analyzed by scRNA-seq to obtain gene expression of each cell, and then cells are clustered as different cell types by using cell markers. (**b**,**d**) Curated ligand-receptor databases are created by collecting ligand-receptor information from the literature and already published databases. (**e**) Cell-cell interactions (CCIs) are predicted by using some scoring functions and thresholds based on the gene expression matrix and cell type annotation. Eventually, CCIs among cell types can be visualized by network, heatmap, or Circos plots, among other visualization tools.

Traditionally, the study of CCIs was limited to in vitro experiments that contained one or two cell types and a few selected genes [6]. The advent of single-cell RNA sequencing (scRNA-seq) [7,8] has opened up the possibility of inferring cell-cell communication through the coordinated expression of all ligand-receptor pairs between single cells. The current state-of-the-art methods for CCI prediction make use of scRNA-seq data and ligand-receptor (L-R) databases, where scRNA-seq data provides gene expression of each cell as an indirect measure of their protein expression, whereas L-R databases give information about potential L-R interactions. Thus, CCIs can be found by measuring the expression level of ligands from source cells and receptors from target cells (Figure 1). Multiple tools for CCI prediction have been published, using different L-R databases, computational methods to estimate expression coordination, and tools for analysis and visualization. Some of these tools, such as CellPhoneDB and CellChat, have already been applied in diverse biomedical studies [5,9,10].

Given the increase on the number of CCI prediction tools, it is important to quantitatively assess their performance, speed, and usability, among other features [11]. Liu et al. performed a comparison of 15 CCI prediction tools [12]. In the absence of a gold standard, the authors compared the CCI predictions to spatial transcriptomics data. Dimitrov et al. built the LIgand-receptor ANalysis frAmework (LIANA), a resource that evaluates scRNA-seq data through five prediction tools, two additional prediction methods, and their ensemble, as well as a compendium of 16 different L-R databases. They compared all possible method-database combinations but also lacked a gold standard and, therefore, their report focused on the overlap between the highest ranked predictions (which is low), robustness to noise (adequate), and enrichment of CCIs among spatially adjacent cell types (present for some datasets only) [13]. Shan et al. created a gold standard of 728 pairs of CCIs [14] and performed a benchmark comparison of the tools from LIANA. However, their study was limited to predictions of “source cell-target cell” interactions, and it did not consider the “source cell-target cell-ligand-receptor” model. Therefore, there is still no comprehensive benchmark study of CCI prediction tools using a curated gold standard made of high-quality experimental CCIs. Such a gold standard-based benchmark could offer better recommendations regarding methods and tools [6].

We have performed such a benchmark study of available CCI prediction tools on idiopathic pulmonary fibrosis (IPF) scRNA-seq data. We compared the output of each tool under evaluation to a gold standard of manually curated CCIs, which was especially designed for IPF as well. Then, we evaluated their prediction performance and computational time. Our goal is to go one step beyond previous method comparison efforts in the following ways: (i) generating a manually-curated gold standard for one specific disease (IPF); (ii) going beyond the “source-target” model to the “source-target-ligand-receptor” model; and (iii) building open jupyter notebooks to facilitate usage of each tool and a workflow to easily replicate our performance study. Therefore, we have created a benchmark study that is transparent and reproducible, as well as easy to improve by adding future methods, tools, databases, or scRNA-seq datasets from a completely different disease or biological system. This way, we consider that our work is an improvement over previous efforts in order to find the best tools for CCI prediction.

## 2. Materials and Methods

### 2.1. Tool Selection

We collected tools from the literature containing the keywords ‘cell-cell interaction/cell-cell communication’ and ‘single-cell RNA sequencing’. Some tools were excluded from our benchmark based on the following criteria: (1) not freely available; (2) no code available; (3) required datasets of two cases (disease/control); (4) required more than expression matrix and cell annotation; for example, genes of interest or spatial data; (5) no detailed source-target or ligand-receptor output; and (6) could not be installed or run successfully (including unresolved errors). We evaluated 16 published tools and 9 of them were excluded (all tools and reasons for exclusion can be found in Supplementary Table S1). Therefore, seven prediction tools were qualified for our benchmark (Table 1).

The final set of tools includes tools belonging to all categories in the classification built by Armingol et al. [1]: There are “Differential combination-based tools”, which find the genes that are differentially expressed between cell clusters to then determine CCIs, such as “iTALK”. “Network-based tools”, which build networks and use network properties such as centrality, such as “NATMI”. And “Expression permutation-based tools”, which compute an interaction score for each L-R pair and then evaluate the interaction significance using permutation strategies. Interaction scoring methods include: identifying L-R pairs whose expression is higher than a threshold, computing the product of L-R expression levels (CellPhoneDB, SingleCellSignalR), using a Hill-function-based mass action model (CellChat), or computing correlation of expression of ligands and receptors across multiple samples [6]. Most tools include different types of visualizations including: Sankey diagrams, heatmaps, dot plots, Circos plots, bipartite networks, alluvial plots, and others. A summary of each tool’s visualization capabilities can be found at Supplementary Table S2. Some of the tools, such as CellPhoneDB, CellChat, and SingleCellSignalR, generate p-values. All tools in our benchmark predict CCI between cell clusters (SoptSC, a method not included here, works with individual cells). Most methods use pairwise interactions, except for CellChat, which accepts interaction mediator proteins, and CellPhoneDB, which accepts protein complex data. Such methods verify that all complex subunits are simultaneously expressed. Another tool not present in our benchmark, scTensor, reportedly predicts higher-order interactions involving more than two cell clusters, while one of our benchmark tools, scMLnet, includes intracellular interactions in target cells.

We have written open and reproducible jupyter notebooks with tutorials for all the available tools, which can be downloaded from our website (https://github.com/mora-lab/cell-cell-interactions/tree/main/interaction-prediction-tools, accessed on 19 November 2021). In addition, we have also included an ensemble of multiple methods through a tool called LIANA [13]. LIANA allows us to run SingleCellSignalR, CellPhoneDB, CellChat, Connectome, iTALK, and NATMI, together with any of the databases used by each of the tools, and compute a combined ranking. Here we used LIANA v.0.1.12 with default settings, which includes an ensemble of five methods (Connectome, NATMI, CellPhoneDB, SingleCellSignalR, and iTALK) plus an ensemble of five databases (CellPhoneDB, CellChatDB, ICELLNET, connectomeDB2020, and CellTalkDB) [13]. Such an ensemble became the eighth evaluated tool of our study.

### 2.2. Gold Standard

Several authors have pointed out the difficulty in generating gold standards for CCIs and the necessity to appeal to more indirect validation methods [13,14]. Our approach has been to generate a gold standard (i.e., a list of source-target and ligand-receptor interactions) for one specific disease. Such a disease-specific gold standard can be regarded as the biological ground truth when compared to predicted CCIs from tools that have received scRNA-seq data for the same disease as an input [21]. We have chosen idiopathic pulmonary fibrosis (IPF) as our common phenotype for both the gold standard and the input scRNA-seq. IPF is a chronic and progressive lung disease caused by fibroblast proliferation and extracellular matrix remodeling. Although the pathogenesis of IPF is still not completely understood, some experiments have revealed that chemokines such as Transforming growth factor beta 1 (TGFβ1), platelet-derived growth factor (PDGF), vascular endothelial growth factor (VEGF), and fibroblast growth factor (FGF) contribute to IPF through CCIs among epithelial cells, fibroblasts, macrophages, and T cells [22].

We have created an IPF-specific manually curated gold standard based on the literature of pathogenesis of IPF, including source-target and up-regulated ligand-receptor information. In total, we collected 250 CCI pairs among eight cell types (alveolar type 1 cell, alveolar type 2 cell, macrophage, fibroblast, endothelial cell, mast cell, monocyte, and T cell). The gold standard can be found on our website (https://github.com/mora-lab/cell-cell-interactions/blob/main/benchmark-workflow/data/, accessed on 19 November 2021). Cell type ontology identifiers were collected from the Cell Ontology (https://www.ebi.ac.uk/ols/ontologies/cl, accessed on 16 March 2022).

To evaluate our strategy, we compared the number of experimentally-supported disease-related CCIs in our dataset to that in CITEdb. CITEdb includes information on 87 diseases, ranging from 1 to 23 interactions per disease, including zero for IPF, as shown in Supplementary Table S3. In opposition, our gold standard has 250 interactions (source-target-ligand-receptor model) or 49 interactions (source-target model) for IPF only. Therefore, we suggest that our “targeted” approach might be better for benchmarking purposes.

### 2.3. scRNA-seq Data Pre-Processing

We searched for scRNA-seq datasets of IPF at the Gene Expression Omnibus (https://www.ncbi.nlm.nih.gov/geo/, accessed on 16 March 2022). Such datasets were required to have both the expression matrix and cell type annotation (if not, their articles should provide information of cell type markers). We found four datasets (GSE122960 [23], GSE128033 [24], GSE135893 [25], and GSE136831 [26]) with 56 IPF samples in total (Supplementary Table S4). We downloaded processed data from datasets GSE135893 and GSE136831 as they provided Seurat objects that already had meta-data of cell types. Datasets GSE122960 and GSE128033 only provided the raw expression matrix, so we downloaded and processed the expression matrix following the standard Seurat procedure [27], i.e., normalize, integrate and scale data, run PCA analysis, find neighbors and clusters, and annotate cell type with marker information from their paper. We only kept cell types that exist in the gold standard. Finally, all datasets were divided into subsets by their sample metadata. For convenience, all datasets were stored as Seurat objects, with metadata cell.type as active.ident to fit all R prediction tools. Such Seurat objects were transformed into matrix and metadata files for the Python tools CellPhoneDB and NATMI. The code corresponding to this data pre-processing workflow can be found at our website (https://github.com/mora-lab/cell-cell-interactions/blob/main/benchmark-workflow/R, accessed on 19 November 2021).

### 2.4. Benchmark Workflow

#### 2.4.1. Run CCI Prediction Using All Tools, and Collect Prediction Results and Computation Time Information

We ran all tools, for each sample, using their default parameters. Regarding thresholds, CellChat, scMLnet, and CellPhoneDB were set to choose interactions with prediction *p*-value < 0.05; SingleCellSignalR includes an index called *LRscore*, which identifies interactions when the score is larger than 0.5; NATMI identifies interactions when detection rates of ligands and receptors are higher than 0.02. Since iTALK and CCInx list all possible interactions with no specific threshold recommended, we chose a number of interactions equal to the maximum amount of predicted interaction pairs among sample outputs from the other five tools. We applied the same rule of thumb to the ranked results from LIANA.

The total number of cells in our study is 138,248. Therefore, that was the maximum number of cells tested during our computations of processing time. The processing time for R tools was calculated using the function proc.time(), while Python tools used the bash command time (only prediction processes were counted). At the same time, cell counts of each dataset were extracted to generate a plot of processing time vs. cell count. Note that not all data could successfully be predicted by all tools. Unresolved error outputs were ignored whereas outputs with 0 predicted interactions were regarded as the sample data not having any significant interaction. To distinguish between the two cases during time processing computations, we wrote functions that produce NA as processing time result for “error outputs”, while extracting the real processing time for “0 interaction outputs”.

#### 2.4.2. Convert Different Outputs to a Unified Format

After prediction, all interaction outputs from both R and Python tools were transformed into the format ‘source-target-ligand-receptor’, except for CellPhoneDB, whose outputs do not provide specific role information of protein A and B. Therefore, interactions predicted by CellPhoneDB were considered as ‘source-target-ligand-receptor’ or ‘target-source-receptor-ligand’ in the following steps. The output of scMLnet consists of ligand-receptor, receptor-TF, and TF-target gene information, but we only kept ligand-receptor pairs for later analysis. Since the databases of CellChat and CellPhoneDB include not only single proteins but also complex information, we separated all pairs from the output that include a complex into pairs of single proteins. For example, the TGFB1-TGFBR complex was divided into TGFB1-TGFBR1 and TGFB1-TGFBR2.

#### 2.4.3. Build Set of All Possible Interaction Pairs

For statistical purposes, we created a set of “all possible pairs”, defined as all possible source-target-ligand-receptor combinations derived from all cells and proteins from the gold standard. To that set, we added the set of all source-target-ligand-receptor interactions from prediction outputs of all tools and removed all duplicated pairs.

#### 2.4.4. Calculate True and False Positives, and True and False Negatives

Prediction outputs for each sample were compared with the gold standard and the set of all possible pairs. We computed the following metrics: True positives (*TP*), which are interactions that appear in both the prediction and the gold standard; false positives (*FP*), which are interactions that appear in the predictions but are not present in the gold standard; true negatives (*TN*), which are interactions that appear in the “all possible” set but do not appear in the predictions or gold standard; and false negatives (*FN*), which mean interactions that appear in the gold standard but are not present in the predictions. It is important to note that our *TN* data is, essentially, a subset of CCIs among our set of cells, ligands and receptors that: (i) has never been reported in the literature, (ii) has been reported in contexts different to IPF, or (iii) might be reported but were missed by us during the construction of the gold standard. There are other approaches to define negative data, such as negative interactions generated from sequence-based computational methods [28] or from high-throughput protein-protein interaction methodologies such as Y2H [29]. Both of them could be used to extract sets of non-interacting ligands and receptors; however, none of them is specific to the cell-cell interaction pairs under our analysis. For CellPhoneDB, both cellA-cellB-protA-protB and cellB-cellA-protB-protA pairs were compared with the gold standard.

#### 2.4.5. Compute Performance Metrics

We studied five different metrics: Precision, Sensitivity, Specificity, *F*1-score, and MCC. Precision determines what percentage of our predictions were in the gold standard and it is useful when we need to control the false positives. It was calculated by:

(1) $$Precision=\frac{TP}{\left(TP+FP\right)}$$

Sensitivity determines what percentage of the gold standard we predicted and it is useful when we need to control the false negatives. It was calculated by:

(2) $$Sensitivity=\frac{TP}{\left(TP+FN\right)}$$

Specificity determines what percentage of the false interactions (*TN* + *FP*) was correctly not predicted to exist (*TN*). It was calculated by:

(3) $$Specificity=\frac{TN}{\left(TN+FP\right)}$$

The *F*1-score is the harmonic mean of precision and sensitivity, and illustrates the quality of positive CCI detection. *F*1-score was calculated by:

(4) $$F1=\frac{2TP}{\left(2TP+FP+FN\right)}$$

Matthews correlation coefficient (*MCC*) considers both positive and negative CCIs. *MCC* was calculated by:

(5) $$MCC=\frac{TP*TN-FP*FN}{\sqrt{\left(TP+FP)(TP+FN)(TN+FP)(TN+FN\right)}}$$

In addition, we plotted Precision-Recall (PR) curves. A PR curve is a plot of the precision versus the sensitivity (also called recall) for different thresholds of a given tool. Usually, there is a trade-off between precision and recall; therefore, the best performing tool is the one with the largest area under the curve (AUC).

#### 2.4.6. Hardware and Software

The benchmark workflow ran under an Oracle’s VirtualBox virtual machine (VM). The hardware included an Intel Xeon(R) Bronze 3104 CPU @ 1.70 GHz × 4 with 64 GB RAM, while the OS was Ubuntu 20.04.5 LTS 64-bit. All the scRNA-seq datasets, the gold standard, the seven tools, and the additional R functions, were installed in the VM where the study was performed. The whole workflow was written and ran in a jupyter notebook that displays both the code and the results. The notebook can be found in our website (https://github.com/mora-lab/cell-cell-interactions/tree/main/benchmark-workflow, accessed on 19 November 2021).

**Figure 2:**
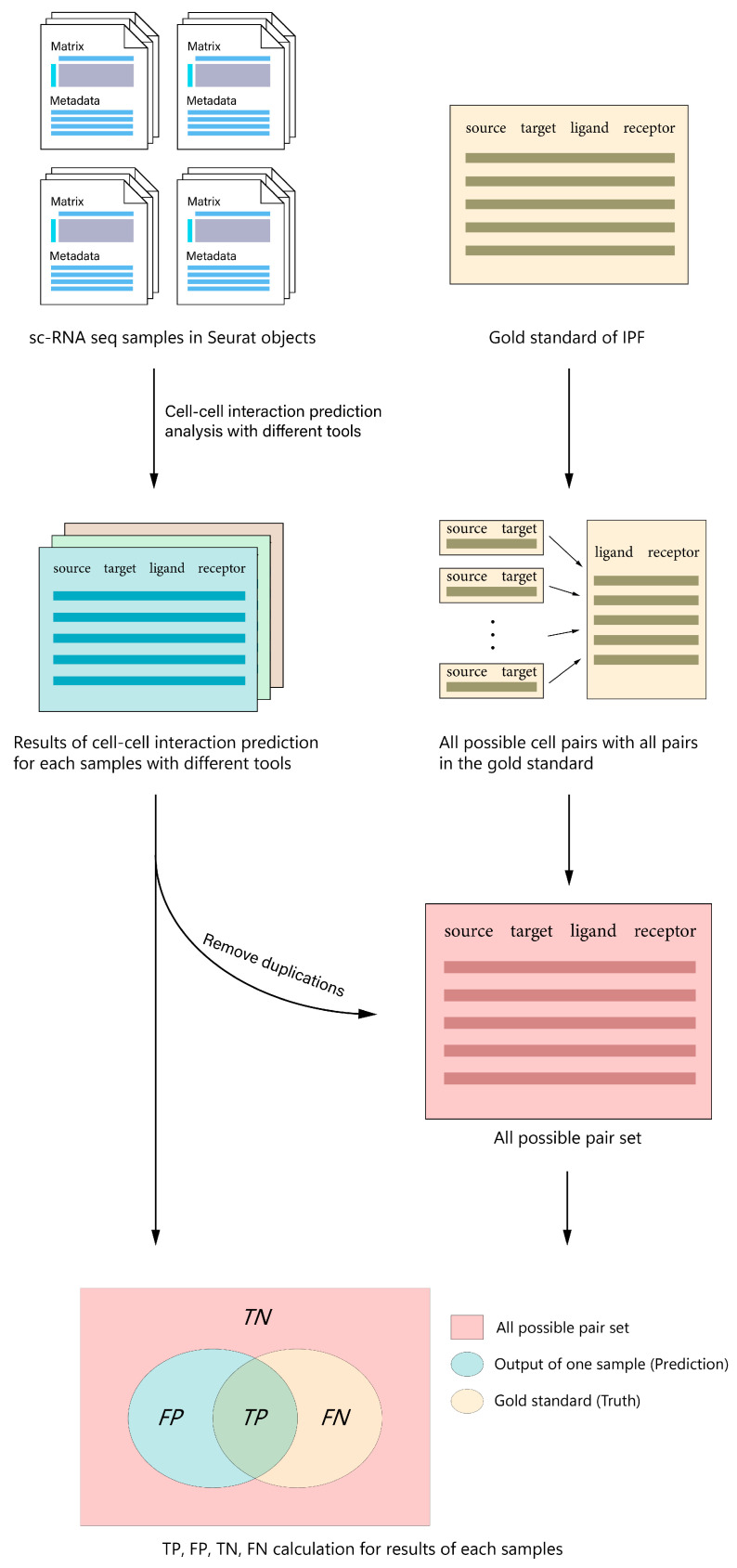
General workflow of our benchmark. CCI analyses were run for each scRNA-seq dataset with all seven tools plus LIANA to create prediction outputs following the “source-target-ligand-receptor” format. All outputs were combined with all possible L-R pairs in the gold standard to create a set of “all possible” pairs. Finally, each prediction output was compared with the gold standard as well as the “all possible” set to calculate *TP*, *FP*, *TN* and *FN*, which are needed to compute precision, sensitivity, specificity, *F*1-score, and *MCC*.

A summary of the workflow can be seen in Figure 2.

## 3. Results

### 3.1. General Setup

**Figure 3:**
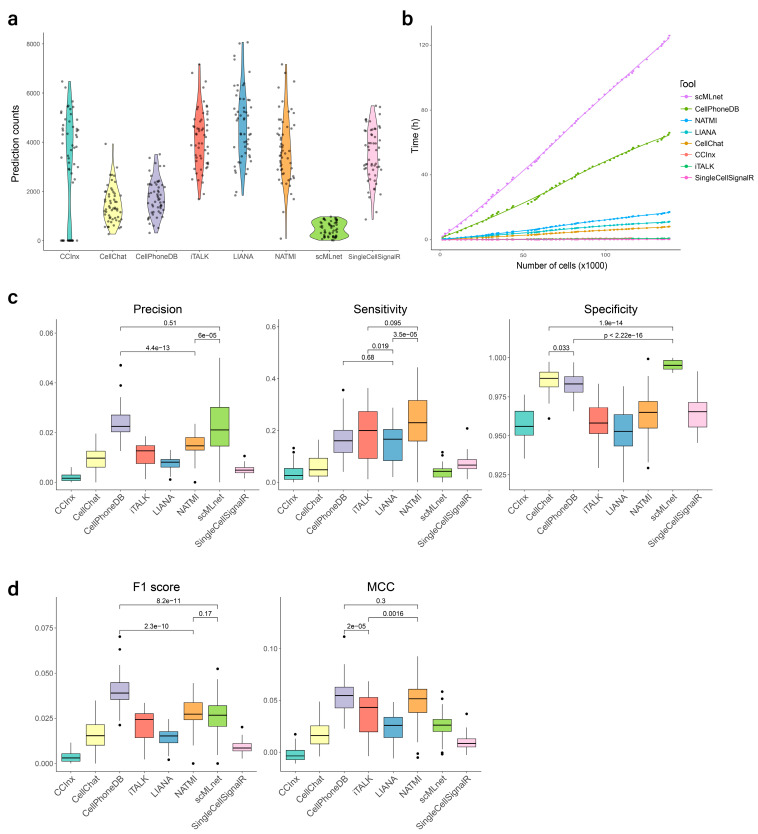
Results of the benchmark study under the STLR (source-target-ligand-receptor prediction) model. (**a**) Predicted CCI pair counts for the seven tools and LIANA’s ensemble. (**b**) Computation time for all CCI prediction tools. (**c**) Precision, sensitivity, and specificity for all CCI prediction tools. (**d**) *F*1-score and MCC for all CCI prediction tools.

**Figure 4:**
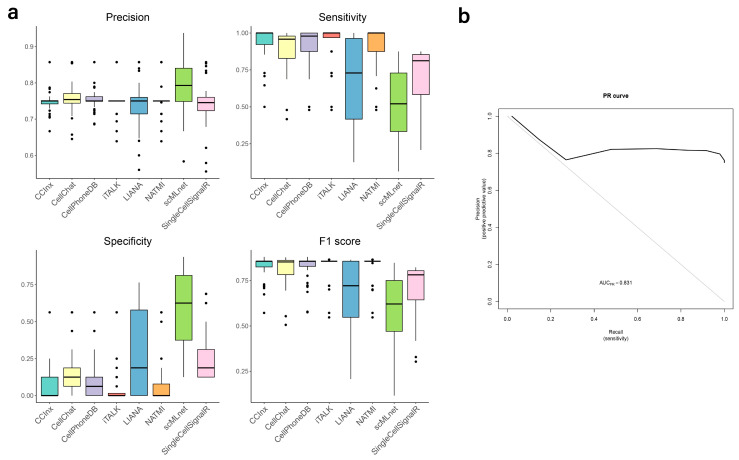
Results of the benchmark study under the ST (source-target prediction) model. (**a**) Precision, sensitivity, specificity, and *F*1-score for all CCI prediction tools. (**b**) Precision-recall curve for CellPhoneDB.

We evaluated every tool’s performance following two different CCI representation models. (i) The STLR model, which means including the source, target, ligand, and receptor for each CCI. Both our gold standard and the software predictions were converted to the STLR model. (ii) The ST model: The ST (source-target) model modifies the STLR tables by counting all the L-R interactions for each cell pair, leaving only cell-cell information. The ST model has been used in a previous benchmark attempt whereas, to our knowledge, the STLR model has not been used. The tool benchmark results for the STLR and the ST models appear in Figure 3 and Figure 4, respectively.

### 3.2. Predicted Interaction Counts

In total, we collected 56 scRNA-seq datasets from IPF patients and 250 CCI pairs from eight cell types in our literature-based manually-curated IPF gold standard. Figure 3a compares the amount of CCIs predicted by the seven tools and LIANA-ensemble from the same input data. During CCI prediction, undetermined errors occurred in 16 samples running CCInx, which were excluded from this part of the analysis. Under the STLR model, scMLnet predicts the smallest number of interactions, although we found that errors happened when sender and receiver cells were the same (autocrine prediction). On the other hand, iTALK, LIANA, and NATMI showed the largest number of predictions. When those predictions are collapsed into the ST model, there is a total of 64 possible CCIs only (given that our gold standard includes eight cell types). We observe that most tools predict all 64 possible interactions in multiple samples, while our gold standard only contains 49 CCIs under this model.

### 3.3. Processing Time

As the technology of scRNA-seq develops, the amount and size of scRNA-seq data are rapidly increasing. Therefore, the speed of a tool needs to be taken into account. We measured the computation time of all seven tools and LIANA-ensemble for different amounts of cells. Figure 3b shows how the speed of the tools varies with cell counts. Reasons for the massive discrepancies between tools might include the differences of methods, algorithms, or prediction depth. SingleCellSignalR, iTALK and CCInx were the fastest prediction tools in our benchmark, followed by CellChat, whereas CellPhoneDB and scMLnet used considerably more time. Regarding scMLnet’s low speed, the fundamental reason is that the tool follows four steps: ligand-receptor prediction, receptor-transcription factor prediction, transcription factor-target gene prediction, and multilayer network construction, which makes it slower than all other tools. We can also see that LIANA’s ensemble is faster than some individual methods, which could have been due to LIANA being a more efficient implementation of some of those methods, or to the fact that LIANA is using older versions of their L-R databases.

### 3.4. Evaluation of Prediction Performance

Under the ST model (source-target prediction only), scMLnet showed the best precision and specificity, but also the worst sensitivity. In contrast, CCInx, CellChat, CellPhoneDB, iTALK, and NATMI, all showed a good precision, with very high sensitivity and very low specificity. Therefore, according to the *F*1-score, the above-mentioned five tools showed the top performance (Figure 4a). In addition, we show that the PR curve for CellPhoneDB (sample 022I from GSE136831) produces an AUC = 0.83, which can be interpreted as a good predictor across threshold values (Figure 4b). Under the STLR model (source-target-ligand-receptor prediction), CellPhoneDB, scMLnet, and NATMI performed better regarding precision, while NATMI, iTALK, LIANA, and CellPhoneDB showed higher sensitivity. For specificity, which is related to true negatives, scMLnet, CellChat and CellPhoneDB achieved higher scores than other tools. It is important to highlight that precision was low for all tools under this model, whereas all tools showed high specificity (Figure 3c). Figure 3d shows that CellPhoneDB, NATMI and scMLnet have the best *F*1-scores, while CellPhoneDB, NATMI and iTALK performed better regarding *MCC*.

The difference in results between source-target prediction and source-target-ligand-receptor prediction deserves further analysis. Under the ST model, as stated before, the tools predict all possible CCIs, not only the true ones, and the number of all possible interactions is close to the number of true ones, rewarding over-prediction and allowing high values of the performance metrics. In addition, we observe that some of those source-target interactions do not include the correct L-R pairs, thus changing FPs into TPs. Therefore, the entire approach could be misleading. On the other hand, under the STLR model, which is a more accurate representation of a CCI, our gold standard contains 250 true CCIs, leading to more than 100,000 possible CCIs (see Section 2). Also, the tools usually predict a few thousand significant interactions, which is much more than the interactions in our gold standard. As a result, the STLR model presents a high imbalance, with *FP* >>> *TP* and total-negatives >>> total-positives. Such an imbalance is responsible for its low values in precision and sensitivity. There are strategies to address the imbalance, which we will discuss later (Figure 3 shows the tool performance values before any technique to address the imbalance is applied). Despite the problems explained, Figure 3 still allows us to identify performance differences between the tools. In summary, we found that, under our settings, predicting all possible ST combinations is enough to obtain excellent metrics under the ST model. We expect that, the more complete the gold standard and the more targeted the study (focused on a specific tissue or location), the more probable is that predicting all cells interacting to each other will give good performance results. Under these conditions, the ST prediction problem becomes trivial, whereas the STLR prediction problem remains a complex one. From this point on, we focus on the STLR model.

### 3.5. Validation of Predictions

In order to verify the ability of the tools to generate new knowledge, we collected the most frequently predicted CCIs among samples by both scMLnet and NATMI under the STLR model (Supplementary Tables S5 and S6). scMLnet predictions were enriched on CCIs related to lung injury. We focused on the “Endothelial-Macrophage-IGF2-IGF2R” interaction, as IGF2 has been reported to affect the inflammatory phenotype of macrophages [30] and it is increasingly expressed in fibrosis diseases [31], which makes this prediction likely to be true. Additional experiments should be performed to validate such interactions between endothelial and macrophage cells in the IPF lung. Regarding NATMI, several of the top CCIs were related to angiogenesis. We focused on the “Endothelial-Mast-IL33-IL1RL1” interaction, as IL33 activation by mast cells plays a significant role in airway inflammation, especially asthma [32]; therefore, we can raise the question of whether IL33 takes part in the pathogenesis of IPF as well. None of the two CCIs mentioned above were found in our literature review (and, therefore, in our gold standard), which indicates the possibility of finding new potential mechanisms using CCI prediction tools.

## 4. Discussion

### 4.1. Tool Recommendations

Different tools could be recommended depending on the user’s goals. When there is a need to control for false positives (e.g, a research focused on a limited number of targets), we prefer tools with high precision and specificity, such as CellPhoneDB and scMLnet. When there is a need to control for false negatives (e.g., a research that is trying to explore all possibilities no matter if some of them are less reliable than others), we prefer tools with high sensitivity, such as NATMI and CellPhoneDB (Figure 3c). If time is an important constraint, CellChat is the fastest tool with high specificity, whereas iTALK is the fastest tool with high sensitivity (Figure 3b). Finally, if we just want an all-round good performance, CellPhoneDB and NATMI are the best tools according to *F*1-score and MCC (Figure 3d). Interestingly, the ensemble of the tools does not represent an improvement over the best performing tools. Even using a comprehensive reference database, the effect of the poor performer methods seems to affect the quality of the ensemble.

In addition to technical features, we have also observed that we should consider other characteristics for tool selection such as: user friendliness, visualization capabilities, and additional analyses. Compared to some R tools, which need coding skills, Python tools like CellPhoneDB and NATMI are easy to use through simple command lines and parameters. Tools like CellChat, iTALK and CellPhoneDB provide various methods for visualization such as chord plots, networks, and bubble plots, which are useful for interpretation of complex CCIs. Finally, in addition to L-R interaction prediction, tools such as CellChat and scMLnet provide complementary information. CellChat can perform further communication analysis such as identification of signaling roles for cells and discovering dominant CCI patterns, while scMLnet can provide more information about receptor-transcription factor and transcription factor-target gene interactions, giving us a multilayer network of cell-cell interactions. All in all, tool selection depends not only on performance but also on project design, intended depth of exploration, coding ability, and hardware configuration, among others.

### 4.2. Comparing Our Study to Other Benchmarks

Our approach differs from other benchmarks attempts such as Dimitrov et al. [13] and Shan et al. [14]. Dimitrov et al. [13] compared 16 databases and seven methods plus the consensus among the methods. They built LIANA, which is an open interface to all methods and databases that decouples each method from its database, allowing the user to compare the combinations of all seven methods with all 16 databases. They also created OmniPath, a database that integrates all 16 resources under study plus a few more. Such resources combine experimental and computational interactions with different degrees of reliability. The authors explain that, due to the lack of a gold standard, they assessed both tools and databases through indirect methods. Regarding the databases, they found varying degrees of overlap as well as bias toward specific terms depending on the resource. They highlight the need for larger curation efforts. Regarding the methods, they applied each method-database pair to six transcriptomic datasets and evaluated the agreement between methods, and the agreement with spatial adjacency, cytokine activity, and receptor protein abundance data. The results showed a low overlap between the highest ranking interactions among the methods but also a general agreement between prediction methods and the other types of data. As they lacked a gold standard, they did not make any specific method recommendations.

Shan et al. [14] created CITEdb, a database that contains 728 manually-curated human cell-cell interactions across 204 different “physiological contexts” (organs, diseases, or disease phases), including metadata such as tissue, experimental approach, and biological functions. The authors specify that the most frequent “contexts” in the database are immune response, bone microenvironment, carcinogenesis, and breast cancer, while stating that this could be used as a benchmark dataset to evaluate CCI prediction tools. In order to test the quality of the dataset, they used their database in conjunction with LIANA. Using LIANA, CITEdb, and an scRNAseq dataset from melanoma, they found that NATMI and SingleCellSignalR were the best performers. However, the authors acknowledge that the main limitation of the approach is that CITEdb’s benchmark ignores ligand–receptor information and focuses on cell-cell interactions (ST model evaluation).

Similar to Shan et al., we went for the “gold standard” approach. However, we abandoned the idea of including many contexts (which leads to mixing evidence from dissimilar contexts and to some contexts containing very few interactions) and replace it with an in-depth coverage of one specific disease. It is not clear how having a gold standard built with little amounts of data for each one of 204 different systems can represent any of those 204 systems when evaluating one specific scRNA-seq dataset. In opposition, our gold standard contains only interactions related to our scRNA-seq dataset and that makes the evaluation more robust. We highlight that, despite our differences, both our benchmark and Shan et al.’s agreed on NATMI being one of the best performers, but we disagree on SingleCellSignalR, which is a good performer under their settings but a poor performer under ours. Similar to Dimitrov et al., we made our benchmarks available for reproducibility but, in our case, we built multiple jupyter notebooks and a virtual machine instance, which allows every researcher to repeat, re-use, and improve our study.

Despite our efforts, our benchmarking approach (STLR model) still presents several limitations: First, our results show low precision of all tools, or the fact that most of the predicted CCIs are not in the gold standard. This calls for experimental validation in order to determine if this is a common problem of the tools or if we are discovering multiple new CCIs that can be added to our gold standard. Second, all tools show medium-to-low sensitivity, or the fact that most of the gold standard was not predicted. This could be a deficiency of the tools but also a result of our methodology: samples could be highly heterogeneous, including patients with different co-morbidities or disease stages (IPF is roughly classified as mild, severe, early, and advanced) and, therefore, no sample will follow all the CCIs from the gold standard. In addition, we computed processing time for all the tools up to 138,248 cells, which is the total number of cells in this study. However, the number of cells in published scRNA-seq datasets is becoming increasingly large. In the future, the tools will also need to prove their efficiency in front of much larger numbers of cells.

The gold standard can be improved regarding quality and quantity. One way of improving the quality of our gold standard is adding disease stages and co-morbidities. Regarding quantity, we can collect CCIs with automatic methods such as text mining followed by manual curation. For the tools that predict CCIs between disease and control groups, the gold standard can be improved by adding not only up-regulated but also down-regulated CCI information. We also observe that our gold standard was built with IPF-specific interactions from the disease mechanism literature; however, there are also “ubiquitous” CCIs to all lungs, sick or healthy, which could also be predicted by the tools and, therefore, should be added to the gold standard as well.

The data imbalance under the STLR model must also be addressed. Some of the ways to address the data imbalance include generating more positives or undersampling the negatives. More importantly, our definition of “true negatives” leads to low numbers of CCIs under the ST model and very large numbers under the STLR model, being one of the culprits of the imbalance. Therefore, an additional option is changing our definition of “true negatives” to experimentally found true negatives. As discussed in “Methods”, we have found a few available datasets of negative protein-protein interactions but we don’t know of any existing dataset including negative source-ligand-receptor-target information.

Finally, it is difficult to say if our results regarding best tools are limited to IPF or can be generalized. We have worked under the assumption that our software recommendations should be generalizable as long as we guarantee that we have a “complete” gold standard of a disease, paired to scRNA-seq data of the same disease, instead of a gold standard made of pieces of mechanisms that do not fully reproduce any single disease. However, it is also possible to think that the choice of disease affects the final method recommendation. Some diseases can have more or less interactions, higher or lower expression levels of proteins, higher or lower levels of multi-protein interactions, and each of those patterns might be easier to detect with different methods. This is a discussion that cannot be solved until a comprehensive gold standard covering multiple diseases/phenotypes and cell types, with a high coverage of each disease and a high quality of interactions, is created.

### 4.3. General Limitations of the Tools and Alternatives to the scRNA-seq Approach

The fact that most tools generate many FPs and few TPs can be partly blamed on the benchmark and partly on the tools themselves. Current tools share multiple limitations that have already been discussed in previous reviews and benchmarks, including:

(1) The fact that neither up-regulation nor expression correlation of the “communicating” molecules necessarily imply that cells are interacting [6].
(2) Communication and structural interactions mainly occur to the protein level, but we use sc-transcriptomics data because sc-proteomics technologies still don’t have the same level of development. However, the assumption that RNA levels reflect protein levels of ligands and receptors is not entirely correct. RNA and protein levels can differ due to multiple post-transcriptional and post-translational processes [1].
(3) Protein-protein interactions are dependent on protein concentration, which is something that most current methods cannot evaluate [1].
(4) Protein abundance is not enough to infer protein-protein interaction strength. Another aspect to consider is the existence of post-translational modifications such as glycosilation. Many ligands and receptors are glycoproteins and glycosylation changes their affinity; therefore, glycomic and other *omic* data could be added under a multi-omic approach [1,6].
(5) All databases are highly incomplete and biased towards some areas of interest.
(6) New methods should consider the temporal aspect of cell-cell communication, i.e., the change in protein levels and communication patterns over time [1,6].
(7) The previous tools do not consider that cellular communication can also be metabolite-mediated. However, recent papers include detailed reviews of metabolite sensing and signaling [33], single-cell mass spectrometry studies of the metabolic profiles of cell-cell interactions [34], and tools for predicting metabolite-mediated CCIs using scRNA-seq data (based on the expression levels of the metabolite-producing enzymes and the metabolite sensors) [35].
(8) Communicating proteins are limited to ligands and receptors. In the future, proteins involved in juxtacrine interactions, such as cell adhesion proteins and gap junctions, extracellular matrix proteins, endocrine signals, and other proteins involved in cell communication, should also be included [13].
(9) Some CCIs are not as simple as one source, one ligand, one receptor, and one target, but they form complex communication pathways instead.

In addition, the tools follow a multiplicity of approaches and there is no unified decision criteria and decision thresholds to predict if a CCI exists. Current tools use from customized scores to *p*-values to no-threshold suggestions. Future implementations should always include decision thresholds that the user is able to manipulate and are not hard-coded in the software, and decision criteria should not be in the form of customized scores with ad hoc thresholds but they should be in terms of the probability of the CCI to be true, which would allow an easier interpretation and comparison.

Those limitations demonstrate that there is still room for improvement in the methods used to predict cell-cell interactions.

On top of that, recent experimental methods have appeared as alternatives to the whole scRNA-seq approach. Such methods have been recently reviewed [36]. They include microscopy-based imaging methods, proximity-based chemical tagging, and functional-based exploitation. Microscopy-based methods include: Transference of GFP (or other fluorophores) from the source cell to the target cell, as well as fluorescence protein reconstitution reports (interaction of two complementary fluorescence protein fragments). Chemical tagging of cell interactions include: (i) contact-dependent tagging, which are technologies that require proximity for a tag from an enzyme on the source cell surface to be transferred to an acceptor on the target cell. (ii) contact-independent tagging, such as technologies where tags diffuse from a catalyst-loaded cell to the interacting cell, or technologies using photocatalytic methods. Finally, functional exploitation methods refer to the application of cell engineering approaches to achieve gain or loss of protein function, such as GFP-based touching nexus (G-baToN) [37] and synNotch [38]. synNotch consists of an engineered Notch receptor that binds to the ligand of interest, as well as a transcription factor that activates expression of proteins such as fluorescent proteins, in response to the ligand-receptor interaction [38]. All the previous strategies are a more direct way of identifying CCIs but lack the high-throughput opportunities that scRNA-seq offers. However, recent methods such as PIC-seq constitute an evolution of the scRNA-seq approach [39]. PIC-seq uses a mild tissue dissociation strategy to preserve some cell structures that are destroyed by common tissue dissociation methods. Such cell aggregates, which have been called PICs (physically interacting cells), are then sequenced using scRNA-seq [39].

## 5. Conclusions

Benchmark studies are aimed to compare the performance of computational methods by reference to a gold standard and in terms of well-known evaluation metrics [11]. Previous to our study, there have been a few attempts to evaluate CCI prediction tools by comparing the overlap of predicted CCIs among them [12,15]; however, agreement is not necessarily related to truth and, therefore, it is important to design, build, and use reference datasets that can represent the biological ground truth. There has also been an attempt to build a gold standard-based benchmark [14]; however, this work has two important limitations: (i) it does not include all information regarding source, target, ligand, and receptor data, and (ii) their gold standard is spread along multiple different phenotypes with many of them containing very few interactions [14]. We have built a benchmark study that improves the previous attempts and offers more reliable conclusions regarding the performance of the current CCI prediction tools. Using scRNA-seq data of IPF and a curated gold standard of IPF’s CCIs collected from the literature, we have been able to evaluate the performance and speed of seven popular, functional, and comparable CCI prediction tools plus a consensus tool, and provide an open benchmark workflow as well as recommendations for CCI prediction tool selection.

Under our settings, all the tools perform well for source-target CCI prediction but show limitations for source-target-ligand-receptor prediction. In general, current state-of-the-art tools for STLR cell-cell interaction prediction work well with scRNA-seq data, showing high specificity, and offering the chance to explore intercellular relationships among several cell types in a tissue. However, low precision and sensitivity issues point to the need of keep improving the gold standard and the benchmark’s design. From our benchmark, CellPhoneDB was the tool with the best performance by *F*1-score and MCC. However, other tools proved to be useful under different circumstances. For example, for an exploratory project such as building a CCI network, which needs as many predicted CCIs as possible, NATMI would be recommended due to its higher sensitivity. For a project looking after one or two specific CCIs, scMLnet would be more adequate. When having a large scRNA-seq dataset and limited computational power, tools such as iTALK might be a good choice.

However, we have found an imbalance between the massive amount of currently available predictions and the limited amount of experimentally validated interactions for specific “source cell–ligand–receptor-target cell” combinations. We need studies that experimentally validate all the predictions that are currently generated and we have discussed in this paper some of the technologies that could be used with that purpose, such as microscopy-based imaging, proximity-based chemical tagging, and functional-based exploitation methods. As a consequence of such an imbalance, current CCI prediction tools have been validated and evaluated on indirect, incomplete, or biased datasets. Therefore, more work is expected from both the tool side and the experimental validation side.

In this paper, we have discussed the many directions in which CCI prediction tools could be improved and, therefore, we expect that more benchmarks for new tools will be needed in the future. For reproducibility and extensibility, all analysis and visualizations in our study were written and run using a jupyter notebook workflow that is open and freely available online. Relevant code and data are also provided, making it possible for other researchers to evaluate their methods using our workflow. We also provide a virtual machine instance with all software installed for even more convenience. We expect that researchers who have new methods or tools for CCI prediction can download our workflow as well as all functions and data, prepare a function following our example to predict CCI, transform their output into a *source-target-ligand-receptor* format, and record the processing time. Our code will produce the plots comparing the evaluation metrics of all the tools. In addition, the user can also test new scRNA-seq datasets or even new gold standards with our workflow by simply replacing the corresponding files. This way, we have built a framework for comparing methods that still do not exist.

Cell-cell interactions are important for understanding disease development and progression, as disruptions in cell-cell interactions can lead to the development of cancer and other disorders. In the future, knowledge of cell-cell interactions might also be useful for predicting and manipulating cell-interaction-based phenotypes. For example, genetic and cell engineering modifications that result in the addition or removal of communication pathways and, consequently, the associated phenotype [1]. As a consequence, we expect the importance and interest on cell-cell interaction prediction, as well as the need for better prediction tools, to keep growing with time.

## Figures and Tables

**Table 1:** CCI prediction tools in our study.

Tool	Language	Method	Database	Output	Reference
CellChat (v.1.1.3)	R	CCI probabilities are calculated using the law of mass action, based on average expression of ligands/receptors by cell groups and cofactors	Curated ligand/receptor database including subunits and cofactors	CellChat object containing all the inferred CCIs with their probabilities	Jin et al. [15]
iTALK (v.0.1.0)	R	CCIs are identified by differentially expressed ligands/receptors between clusters	Manually curated ligand/receptor database	All CCIs with their mean ligand/receptor expression	Wang et al. [16]
SingleCellSignalR (v.1.8.0)	R	LRscore, a regularized score, is utilized to assess the confidence in predicted ligand-receptor interactions by controlling false positives	Curated ligand/receptor database with existing sources and manual additions	All CCIs with their regularized LRscore	Cabello-Aguilar et al. [17]
CCInx (v.0.5.1)	R	CCIs are predicted using ligand/receptor expression magnitude to rank nodes	Bader Lab’s ligand/receptor database	Node and edge list of all CCIs	https://github.com/BaderLab/CCInx (accessed on 19 November, 2021)
scMLnet (v.0.1.0)	R	Fisher’s exact test and correlation	Curated ligand/receptor/TF/target database with prior studies and databases	Ligand-receptor, receptor-TF and TF-target interactions	Cheng et al. [18]
CellPhoneDB (v.2.0.0)	Python	CCIs are enriched by empirical shuffling and statistical test	Curated ligand/receptor database (including complex information) with prior studies and databases	Table with information of ligand-receptor pairs by cell pairs, and their *p*-value	Efremova et al. [19]
NATMI	Python	Edge weights of CCIs are calculated by normalized expression level of ligands/receptors	connectomeDB2020 (a database of manually curated ligand-receptor pairs with literature support)	Table of ligand-receptor pairs, their expression level and edge weights	Hou et al. [20]

## Data Availability

Original scRNA-seq data used in this project is available from the Gene Expression Omnibus (https://www.ncbi.nlm.nih.gov/geo/), under ID numbers GSE122960, GSE128033, GSE135893, and GSE136831. Processed datasets for our workflow have been uploaded to: https://zenodo.org/record/6497091. To ensure the transparency, extensibility and reproducibility of our work, we have uploaded the workflow, including all code and our gold standard, to: https://github.com/mora-lab/cell-cell-interactions/blob/main/benchmark-workflow. Finally, a Linux virtual machine instance with all software, data, and notebooks is available from: https://www.zenodo.org/record/8020387. Any future updates to this work will be uploaded to https://github.com/mora-lab/cell-cell-interactions/.

## Supplementary Materials

The following supporting information [13,14,15,16,17,18,19,20,40,41,42,43,44,45,46,47] can be downloaded at: https://www.mdpi.com/article/10.3390/biom13081211/s1, Table S1. Reviewed CCI prediction tools. Table S2. Visualization comparison of reviewed prediction tools. Table S3. Distribution of experimental cell-cell interactions per disease in CITEdb. Table S4. scRNA-seq datasets of IPF used in our study. Table S5. Top CCIs predicted by scMLnet from outputs of all samples. Table S6. Top CCIs predicted by NATMI from outputs of all samples.
