# Supplementary material for: A Comparison of Cell-Cell Interaction Prediction Tools Based on scRNA-seq Data
Source: Biomolecules. 2023 Aug 2;13(8):1211. doi: 10.3390/biom13081211 (PMC10452151; doi:10.3390/biom13081211)
Supplement: Supplementary file 1 [file biomolecules-13-01211-s001.zip › biomolecules-2484775-supplementary.pdf]

biomolecules

MDPI

Supplementary Table S1. Reviewed CCI prediction tools

Tool	Inplementation	Method	Database	Output	Reference	Qualified/ Reason for exclusion
CellChat	R	CCI probabilities are calculated using the law of mass action, based on average expression of ligands/receptors by cell groups and cofactors	Curated ligand/receptor database including subunits and cofactors	CellChat object containing all the infered CCIs with their probabilities	Jin et al. [1]	Qualified
iTALK	R	CCIs are identified by differentially expressed ligands/receptors between clusters	Manually curated ligand/receptor database	All CCIs with their mean ligand/receptor expression	Wang et al. [2]	Qualified
SingleCellSignalR	R	LRscore, a regularized score, is utilized to assess the confidence in predicted ligand-receptor interactions by controlling false positives	Curated ligand/receptor database with existing sources and manual additions	All CCIs with their regularized LRscore	Cabello-Aguilar et al. [3]	Qualified
CCInx	R	CCIs are predicted using ligand/receptor expression magnitude to rank nodes	Bader lab's ligand/receptor database	Node and edge list of all CCIs	https://github.com/BaderLab/CCInx	Qualified
scMLnet	R	Fisher's exact test and correlation	Curated ligand/receptor/TF/target database with prior studies and databases	Ligand-receptor, receptor-TF and TF-target interactions	Cheng et al. [4]	Qualified
CellPhoneDB	Python	CCIs are enriched by empirical shuffling and statistical test	Curated ligand/receptor database (including complex information) with prior studies and databases	Table with information of ligand-receptor pairs by cell pairs, and their p-value	Efremova et al. [5]	Qualified
NATMI	Python	Edge weights of CCIs are calculated by normalized expression level of ligands/receptors	connectomeDB2020 (a database of manually curated ligand-receptor pairs with literature support)	Table of ligand-receptor pairs, their expression level and edge weights	Hou et al. [6]	Qualified
LIANA	R	Robust Rank Aggregation for outputs from different methods	16 CCI inference resources	List containing CCIs predicted with	Dimitrov et al. [7]	Qualified

				different tools and resources		
NicheNet	R	CCIs are inferred based on gene expression and a ligand-target regulatory potential model	Built by integrating prior knowledge on ligand-to-target signaling paths (collected from 57 databases)	Table of ligand-receptor pairs and their interaction score	Browaeys et al. [8]	Samples of control group are required
scTensor	R	CCIs are inferred with tensor decomposition	Curated database (LRBase.XXX.eg.db)	HTML files with ligand-receptor pattern interactions	Tsuyuzaki et al. [9]	Output type is not source-target/ligand-receptor pattern
cell2cell	Python	CCIs are predicted by expression of ligands and receptors and their Bray-Curtis scores	Curated ligand-receptor database	Table with information of ligand-receptor pairs by cell pairs, and their p-value	Armingol et al. [10]	Failed to install
PyMINEr	Python	CCIs are identified by differentially expressed genes or significantly enriched genes	gProfiler tool (Reimand et al., 2016) and StringDB (Szklarczyk et al., 2015)	Table of paracrine/autocrine interactions	Tyler et al. [11]	Unsolved error
SoptSC	R and MATLAB	Signaling probabilities between sender and receptor cells are calculated	Not provided	Table of cell-cell and cluster-cluster interaction probabilities	Wang et al. [12]	No database provided
CellTalker	R	CCIs are detected by differentially expressed genes in each cluster	Database from Ramilowski et al. (Nature Comms, 2015)	Upregulated and downregulated interactions between clusters	https://github.com/arc85/celltalker	Samples of control group are required
CytoTalk	R	Prize-collecting Steiner tree algorithm	Curated database of 1942 manually annotated ligand-receptor interactions	De novo construction of signaling networks between cell types	Hu et al. [13]	Dependency installation issues
CellCall	R	Calculated by the expression value of ligands and receptors	KEGG pathway	S4 object containing a ligand-receptor score between cell types	Zhang et al. [14]	Failed to install
SpaOTsc	Python	Structured and unbalanced optimal transport to recover interactions	Curated ligand-receptor database (Ramilowski et al.)	Table of ligand-receptor and their probabilities	Cang et al. [15]	Spatial data is required

References

1. Jin S, Guerrero-Juarez CF, Zhang L, Chang I, Ramos R, Kuan CH, et al. Inference and analysis of cell-cell communication using cellchat. *Nature communications* **2021**, *12*, 1088
2. Wang Y, Wang R, Zhang S, Song S, Jiang C, Han G, et al. iTalk: An R package to characterize and illustrate intercellular communication. *bioRxiv* **2019**, 507871
3. Cabello-Aguilar S, Alame M, Kon-Sun-Tack F, Fau C, Lacroix M, Colinge J. SingleCellSignalR: Inference of intercellular networks from single-cell transcriptomics. *Nucleic acids research* **2020**, *48*, e55
4. Cheng J, Zhang J, Wu Z, Sun X. Inferring microenvironmental regulation of gene expression from single-cell RNA sequencing data using scmlnet with an application to covid-19. *Briefings in bioinformatics* **2021**, *22*, 988-1005
5. Efremova M, Vento-Tormo M, Teichmann SA, Vento-Tormo R, Hou R, Denisenko E, et al. CellphoneDB: Inferring cell-cell communication from combined expression of multi-subunit ligand-receptor complexes. *Nature protocols* **2020**, *15*, 1484-1506
6. Hou R, Denisenko E, Ong HT, Ramilowski JA, Forrest ARR. Predicting cell-to-cell communication networks using NATMI. *Nature communications* **2020**, *11*, 5011
7. Dimitrov D, Türei D, Garrido-Rodriguez M, et al. Comparison of methods and resources for cell-cell communication inference from single-cell RNA-Seq data. *Nat Commun* **2022**, 13, 3224
8. Browaeys R, Saelens W, Saeys Y, Tyler SR, Rotti PG, Sun X, et al. NicheNet: Modeling intercellular communication by linking ligands to target genes. *Nature methods* **2020**, *17*, 159-162
9. Tsuyuzaki K, Ishii M, Nikaido I. Uncovering hypergraphs of cell-cell interaction from single cell RNA-sequencing data. *bioRxiv* **2019**, 566182
10. Armingol E, Ghaddar A, Joshi CJ, Baghdassarian H, Shamie I, Chan J, et al. Inferring a spatial code of cell-cell interactions across a whole animal body. *PLoS Comput Biol* **2022**, 18, e1010715
11. Tyler SR, Rotti PG, Sun X, Yi Y, Xie W, Winter MC, et al. PyMiner finds gene and autocrine-paracrine networks from human islet scRNA-seq. *Cell reports* **2019**, *26*, 1951-1964, e1958
12. Wang S, Karikomi M, MacLean AL, Nie Q. Cell lineage and communication network inference via optimization for single-cell transcriptomics. *Nucleic acids research* **2019**, *47*, e66
13. Hu Y, Peng T, Gao L, Tan K. CytoTalk: De novo construction of signal transduction networks using single-cell transcriptomic data. *Science Advances* **2021**, 7, eabf1356.
14. Zhang Y, Liu T, Hu X, Wang M, Wang J, Zou B, Tan P, Cui T, Dou Y, Ning L, Huang Y, Rao S, Wang D, Zhao X. CellCall: integrating paired ligand-receptor and transcription factor activities for cell-cell communication. *Nucleic Acids Res* **2021**, 49, 8520-853
15. Cang Z, Nie Q. Inferring spatial and signaling relationships between cells from single cell transcriptomic data. *Nature communications* **2020**, *11*, 2084

Supplementary Table S2. Visualization comparison of reviewed prediction tools

Tool	Network	Circos plot	Heatmap	Bubble plot
NicheNet	Y	Y	Y	Y
NATMI	Y	Y	Y	
iTALK	Y	Y		
scTensor	Y		Y	
CellPhoneDB			Y	Y
SoptSC			Y	
scMLnet	Y			
SingleCellSignalR		Y		
CCInx	Y			
celltalker		Y		
PyMINEr				
CellChat		Y	Y	Y
LIANA		Y	Y	Y

Supplementary Table S3. Distribution of experimental cell-cell interactions per disease in CITEdb

Disease	Source-target model	Source-target-ligand-receptor model
acute inflammation	5	0
acute lymphoblastic leukemia	2	0
allergic contact dermatitis	1	0
Alzheimer's disease	2	0
Asthma	15	0
asthmatic lung	1	0
Atherogenesis	3	0
atherosclerosis	12	1
atherosclerotic plaque	10	19
autoimmune disease	1	0
bone metastatic microenvironment	2	2
breast cancer	22	21
Cancer	9	1
cancer progression	3	0
carcinogenesis	20	0
cardiac disease	1	0
cardiac hypertrophy	3	0
Cholesteatoma	1	0
chronic airway inflammation	1	0
chronic alcohol abuse	1	0
colorectal adenocarcinoma	1	0
colorectal cancer	1	0
COPD	2	0
COVID-19	5	4
cystic fibrosis	1	0
Diabetes	2	0
diabetes mellitus	7	2
diabetes mellitus, type 1	1	0
EBV infection	1	0
gastric cancer	3	0
gastrointestinal stromal tumors	1	0
Glioblastoma	1	0
glioblastoma microenvironment	3	0
Glioma	1	0
head and neck squamous cell carcinoma	10	10
heart failure	2	1
heart injuries	1	0
hepatitis C virus (HCV) infection	1	0
hepatocellular carcinoma	4	0

HIV infection	2	0
human immunodeficiency virus type-1 infection	2	0
Infertility	1	0
influenza, human	3	0
intervertebral disc degeneration	1	0
ischemic injury	3	0
kidney disease	3	0
Leukemia	1	0
liver cancer	3	0
liver fibrotic	6	0
lung adenocarcinoma	3	0
lung cancer	10	0
lung injury	3	0
Melanoma	5	3
metastatic melanoma	13	13
multiple myeloma	1	0
myocardial ischemia?Creperfusion injury	2	0
nasopharyngeal carcinoma	1	0
Neoplasms	7	0
neuroblastoma	2	0
neurodegenerative disease	1	0
non-alcoholic fatty liver disease	5	3
non-small lung cancer	1	0
Obesity	2	0
Osteoarthritis	3	0
ovarian cancer	1	0
pancreatic cancer	1	0
periodontal disease	1	0
Periodontitis	9	1
pituitary tumor	1	0
Plasmodium vivax	5	0
primary liver cancer	1	0
prostate cancer	2	0
Psoriasis	2	0
renal cell carcinoma	1	0
retinal disease	1	0
rheumatoid arthritis	3	5
Schizophrenia	1	0
Silicosis	4	0
squamous cell carcinoma	6	4
stomach neoplasms	1	0
Thrombosis	1	0

tumor microenvironment	11	0
type 2 diabetes	2	0
ulcerative colitis inflamed	2	0
ulcerative colitis non-inflamed	2	0
uterine cellular leiomyosarcoma	1	1
uterus injury	4	0

Supplementary Table S4. scRNA-seq datasets of IPF used in our study

GEO Dataset	Sample Name
GSE122960	IPF_01
	IPF_02
	IPF_03
	IPF_04
GSE128033	SC153IPFLOW
	SC154IPFUP
	SC87IPFLOW
	SC88IPFUP
	SC89IPFLOW
	SC93IPFLOW
	SC94IPFUP
	SC95IPFLOW
GSE135893	TILD001
	TILD006
	TILD010
	TILD015
	TILD028
	VUILD53
	VUILD59
	VUILD60
	VUILD61
	VUILD63
	VUILD64
	VUILD65
GSE136831	010I
	021I
	022I
	025I
	034I
	040I
	041I
	051I
	053I
	063I
	123I
	135I
	138I

145I
157I
158I
166I
174I
177I
179I
209I
210I
212I
214I
221I
222I
225I
228I
29I
47I
49I
59I

Supplementary Table S5. Top CCIs predicted by scMLnet from outputs of all samples

source-target-ligand-receptor	Prediction frequency
Endothelial-Macrophage-CTGF-ITGB2	44
Endothelial-Macrophage-PLAT-ITGB2	42
Endothelial-Macrophage-CYR61-ITGB2	40
Macrophage-Endothelial-IL1RN-IL1R1	39
Endothelial-Macrophage-VCAM1-ITGB2	38
Endothelial-Macrophage-CTGF-LRP1	37
Endothelial-Macrophage-SERPINE1-PLAUR	37
Macrophage-Endothelial-IL1B-IL1R1	37
Endothelial-Macrophage-TFPI-LRP1	36
Endothelial-Monocyte-SERPINE1-PLAUR	36
Fibroblast-Macrophage-C3-ITGB2	36
Endothelial-Macrophage-APP-LRP1	35
Endothelial-Macrophage-PLAT-LRP1	35
Endothelial-Monocyte-CTGF-ITGB2	35
Fibroblast-Macrophage-RARRES2-CCRL2	35
Endothelial-Macrophage-IGF2-IGF2R	34
Endothelial-Monocyte-CYR61-ITGB2	33
Endothelial-Monocyte-PLAT-ITGB2	33
Macrophage-Endothelial-BST1-CAV1	33
Endothelial-Macrophage-F8-LRP1	32

Supplementary Table S6. Top CCIs predicted by NATMI from outputs of all samples

source-target-ligand-receptor	Prediction frequency	Sum of prediction specificity
Tcell-Endothelial-CCL5-ACKR1	50	40.99493279
Endothelial-Endothelial-MMRN2-CLEC14A	46	38.77105524
Fibroblast-Fibroblast-COL3A1-DDR2	48	36.25142374
Endothelial-Endothelial-MCAM-MCAM	44	34.05091879
Fibroblast-Fibroblast-COL1A1-DDR2	47	32.7013714
Endothelial-Fibroblast-MMRN2-CD248	39	32.17917896
Endothelial-Macrophage-CCL14-CCR1	51	31.92938764
Fibroblast-Endothelial-SLIT3-ROBO4	36	31.760152
Endothelial-Mast-IL33-IL1RL1	46	29.414662
Fibroblast-Endothelial-COL1A2-CD93	52	28.99694094
Fibroblast-Endothelial-SLIT2-ROBO4	32	27.62274994
Tcell-Macrophage-CCL5-CCR1	50	26.89195676
Fibroblast-Endothelial-COL1A1-CD93	52	26.34483125
Fibroblast-Endothelial-ANGPTL1-TEK	28	24.75304148
Endothelial-Endothelial-MMRN2-CD93	47	24.64677342
Tcell-Tcell-CLEC2D-KLRB1	35	24.58061868
Tcell-Tcell-KLRB1-CLEC2D	35	24.58061868
Fibroblast-Endothelial-SERPING1-SELP	43	23.84439683
Fibroblast-Monocyte-DCN-TLR2	48	23.82710366
AT2-Endothelial-SFTPA2-CD93	48	23.76355201
